# Mechanism of Inflammatory Associated Impairment of Sperm Function, Spermatogenesis and Steroidogenesis

**DOI:** 10.3389/fendo.2022.897029

**Published:** 2022-04-28

**Authors:** Hiba Hasan, Sudhanshu Bhushan, Monika Fijak, Andreas Meinhardt

**Affiliations:** Institute of Anatomy and Cell Biology, Hessian Centre of Reproductive Medicine, Justus-Liebig University Giessen, Giessen, Germany

**Keywords:** testicular infection, testicular inflammation, autoimmunity, paracrine regulation, oxidative stress, ROS, cytokines, chemokines

## Abstract

Infection and inflammation are relevant entities of male reproductive disorders that can lead to sub-/infertility. Associated damage of the testis of affected men and in rodent models include leukocytic infiltration, edema formation, fibrosis, germ cell loss and reduced androgen levels. Negative effects on spermatogenesis are thought to be elicited by oxidative stress sustained mostly by increased levels of ROS and pro-inflammatory cytokines. Under normal conditions these cytokines have physiological functions. However, increased levels as seen in inflammation and infection, but also in obesity and cancer are harmful for germ cells and impair steroidogenesis. As a summary, there is mounting evidence that the activation of inflammatory pathways is a rather common feature in various forms of male testicular disorders that extends beyond established infectious/inflammatory cues. This mini review will focus on relevant entities and the mechanisms of how a dysbalance of local testicular factors contributes to disturbances of spermatogenesis and steroidogenesis.

## Conditions Leading to Testicular and Epididymal Inflammation and Their Influence on Hormone Levels, Steroidogenesis, Spermatogenesis and Semen Quality

The testis is an immune privileged organ that tolerates the introduction of sperm autoantigens at the onset of puberty without eliciting an inflammatory immune response (1). The testis in mammals evolved multiple strategies to preserve this immunocompromised status, namely, the formation of the blood-testis-barrier (BTB) between adjacent Sertoli cells that secludes most of the developing germ cells from the interstitial compartment where leukocytes reside (1, 2). Besides the BTB the Sertoli cells display important immunoprotective functions that may also contribute to immune privilege. This has been shown when Sertoli cells were co-transplanted with allo- or xenografts thereby prolonging the survival of pancreatic islets (3), hepatocytes (4) and neurons (5) as well as other types of cells (6). Moreover, through Sertoli cells, antigens protected from transcellular leakage by the BTB can egress *via* transcytosis into the interstitial space where antigen-presenting cells (dendritic cells, macrophages) help to maintain Treg tolerance to meiotic antigens. Depletion of the Treg leads to autoimmune orchitis emphasizing the importance of the Sertoli cell-macrophage-Treg axis in maintaining immune privilege (7).

Evidence suggests that immunological and infectious etiologies contribute substantially to male infertility [accounting for 13–15% of cases (2)], a medical and social problem which in total is increasing worldwide (8). The contribution of inflammatory infertility may be underestimated as immune cell infiltration is observed in 20% of testicular biopsies of azoospermic infertile patients (9). Moreover, increased infiltration of immune cells into the testes with concomitant impairment in testicular functions is associated with certain chronic diseases, namely atherosclerosis and cancer (10–12). Given that infection and inflammation are critical drivers of male infertility, we will highlight how these entities can impair the archetypical functions of the male gonad, i.e. spermatogenesis and steroidogenesis.

Local inflammatory conditions of the testis, because of acute infection or inflammatory testicular reactions of unknown origin as well as systemic inflammatory conditions, all can negatively impact spermatogenesis and steroidogenesis. They can do so at the following levels: (a) direct impairment of spermatogenesis, sperm quality and function, e.g. by germ cell death, oxidative stress and impaired mitochondrial activity, (b) disruption of steroidogenesis due to perturbation of the hypothalamic-pituitary-testicular axis, (c) obstruction of the male genital tract or (d) dysfunction of accessory glands (13–15). The following sections will elaborate in more detail on relevant factors and mechanism of disease.

### Bacterial infections

In the clinic, Escherichia coli (E. coli), Proteus mirabilis, Staphylococcus aureus, Streptococcus veridans, Ureaplasma urealyticum, Mycoplasma hominis and Chlamydia trachomatis are commonly isolated pathogens in liquid biopsies of men with genitourinary tract infection (16, 17). Among these bacteria, E. coli and Chlamydia trachomatis are the most clinically relevant pathogens and thus are frequently used in animal studies to mimic the human condition (16). Currently, rodent models propose two routes of infection for these microbes with uropathogenic E. coli (UPEC) reaching the epididymis and testis via ascending canicular infection after injection into the vas deferens. Alternatively, for Chlamydia muridarum, a murine-specific pathovar, macrophages were suggested as a vector as luminal spread from the infection site at the urethral orifice was excluded by vasectomy (18–20). Although not seen as vectors for UPEC, infiltrating monocyte-derived macrophages also appear to be crucial in the immunopathology associated with acute epididymo-orchitis which was convincingly shown in Ccr2^-/-^ mice, which lack blood monocytes due to defective egress from the bone marrow (21).

In clinical practice, epididymitis is almost exclusively of infectious origin. Leukocytospermia is seen often in the acute phase of disease; however, approximately 40-50% of epididymitis patients show persistent impaired semen parameters affecting sperm concentrations, motility and morphology (22). In up to 60% of all cases, the testis is also affected in a combined epididymo-orchitis as follow-up biopsies revealed severe hypospermatogenesis indicated by loss of germ cells in the adluminal compartment of the seminiferous epithelium, massive infiltration of the interstitial and even tubular compartment by immune cells, a thickened lamina propria and interstitial fibrosis. These alterations were accompanied by increased FSH levels (23, 24). Of note, persistent azoospermia in 10% and oligozoospermia in 30% of men suffering from acute epididymitis is detected (15, 22). Interestingly, sperm proteome analysis in patients after recovery from epididymitis (3 months) demonstrated long-term alterations in protein composition (25). Besides changes in the proteome, the glycome of sperm was altered in men with a history of epididymitis as seen by a substantial reduction of sialic acid residues on the surface of spermatozoa (26).

### Viral infections

Several viruses, namely human immunodeficiency virus (HIV-1), Zika virus (ZIKV), Ebola and Marburg viruses as well as the mumps orthorubulavirus (MuV) can infect not only the testes but also the entire male reproductive tract of human and non-human primates through the hematogenous route (14). These viruses silently propagate inside the organ for an extended time. Recent studies suggest that the testicular macrophages are the reservoir for a few viruses and are critical for initiating infection and later dissemination into other testicular cells. For example, the ZIKV colonized the interstitial CD206^+^ testicular macrophages and then spread infection into the seminiferous tubules (27). Similarly, another study demonstrated that the S100A4^+^ macrophages were susceptible to ZIKV infection that facilitated ZIKV dissemination and persistence in the seminiferous tubules (28). After internalizing ZIKV, testicular macrophages skewed towards a pro-inflammatory phenotype and secreted pro-inflammatory cytokines. These disturb the BTB in a paracrine fashion by down-regulating claudin-1 expression and facilitating S100A4^+^ macrophage entry into the seminiferous tubules (28). In contrast to ZIKV, Marburg virus mainly colonized Sertoli cells leading to a disruption of the BTB. In addition, infection with Marburg virus results in increased infiltration of immune cells in the testis, namely CD68^+^ macrophages/monocytes, CD3^+^ T cells and B cells in both the interstitial space and seminiferous tubules leading to spermatogenic cell loss and severe testicular damage (29).

Viral infection alters endocrine, sperm and semen parameters by targeting the male reproductive tract directly and indirectly (systemic). In relation to systemic infections (e.g. influenza), fever could result in increased testicular temperature and subsequent disturbances in spermatogenesis and steroidogenesis by perturbation of the hypothalamo-pituitary-gonadal axis (30, 31). In the context of viral infections, alterations in spermatozoal (count, motility, morphology) and semen parameters (e.g. volume of seminal plasma, viscosity, pH, enzyme concentrations) were reported, in some cases accompanied by orchitis (32–37). Impairment of spermatogenesis could be related to different mechanisms including inflammatory reactions in the reproductive organ, disruption of the testicular cytokine milieu, decreased testosterone production by Leydig cells, disturbances in the paracrine control by somatic cells, change in testicular temperature due to fever and viral replication within cells of the male genital tract. Of note, macrophages, Sertoli cells and germ cells may serve as viral reservoirs [reviewed in (14)]. In chronic viral orchitis, histology of affected seminiferous tubules reveal degeneration of the germinal epithelium accompanied by thickening of the lamina propria, which ultimately may result in complete hyalinization and fibrosis of the tubules leading to the formation of so called “tubular shadows” (38). In Leydig cells, viral replication can lead to decreased testosterone production (39–41) an observation that was reported to be accompanied by changes in LH, FSH or inhibin B levels (32, 33, 36, 37, 41).

### Autoimmunity

Autoimmune orchitis is an inflammation of the testis, where autoimmune reactions against spermatic antigens cause damage to germ cells, and also to testicular somatic cells. It is a rare disease in men with the potential to impede the normal function of the testis. Mutation in the autoimmune regulator (*Aire*) gene results in human autoimmune polyendocrine syndrome APS-type 1 (APS-1), which is characterized by autoimmune reactions in several organs, including the testes (42). This observation is corroborated in *Aire*-deficient mice that reproduced many clinical signs of APS-1 in human (43).

In men, histopathological analysis of testicular biopsies with inflammatory lesions of idiopathic origin show that lymphocytic infiltrates correlate with tubular damage, visible as partial or complete loss of the germinal epithelium, thickening of the lamina propria and tubular fibrosis. These changes are associated with reduced testicular volume and score counts for spermatogenesis, while FSH levels are not increased in these patients (2, 38). Similar histopathological changes are also seen in a mouse model of autoimmune-based epididymo-orchitis (EAEO) elicited by injection of testicular homogenate. Here, the disease can develop progressively up to the formation of granulomas. In rodent EAEO, FSH levels are concomitantly increased, while testosterone levels are reduced. This possibly points to a negative local paracrine influence on Leydig cell steroidogenesis. This assumption is supported by the observation that basal and hCG stimulated production of testosterone is elevated in isolated primary Leydig cells from EAEO rats compared to control. TNF-α abolishes this increase in testosterone [reviewed in (2, 44)].

In addition, systemic low grade inflammatory conditions associated with obesity including complications leading to cardiovascular disease, type 2 diabetes mellitus, malignancy and accelerated aging are connected with alterations in the hypothalamic-pituitary-gonadal axis, poor semen quality and disruption of testicular steroidogenesis. Obesity impacts negatively semen parameters (sperm concentration, motility, viability, morphology) and sperm function (chromatin condensation, DNA fragmentation, apoptosis and epigenetic signatures [reviewed in (45, 46)].

## Inflammatory Disorder Related Mechanisms and Pathways

### Influence of Oxidative Stress on Spermatogenesis and Steroidogenesis

**Figure 1:**
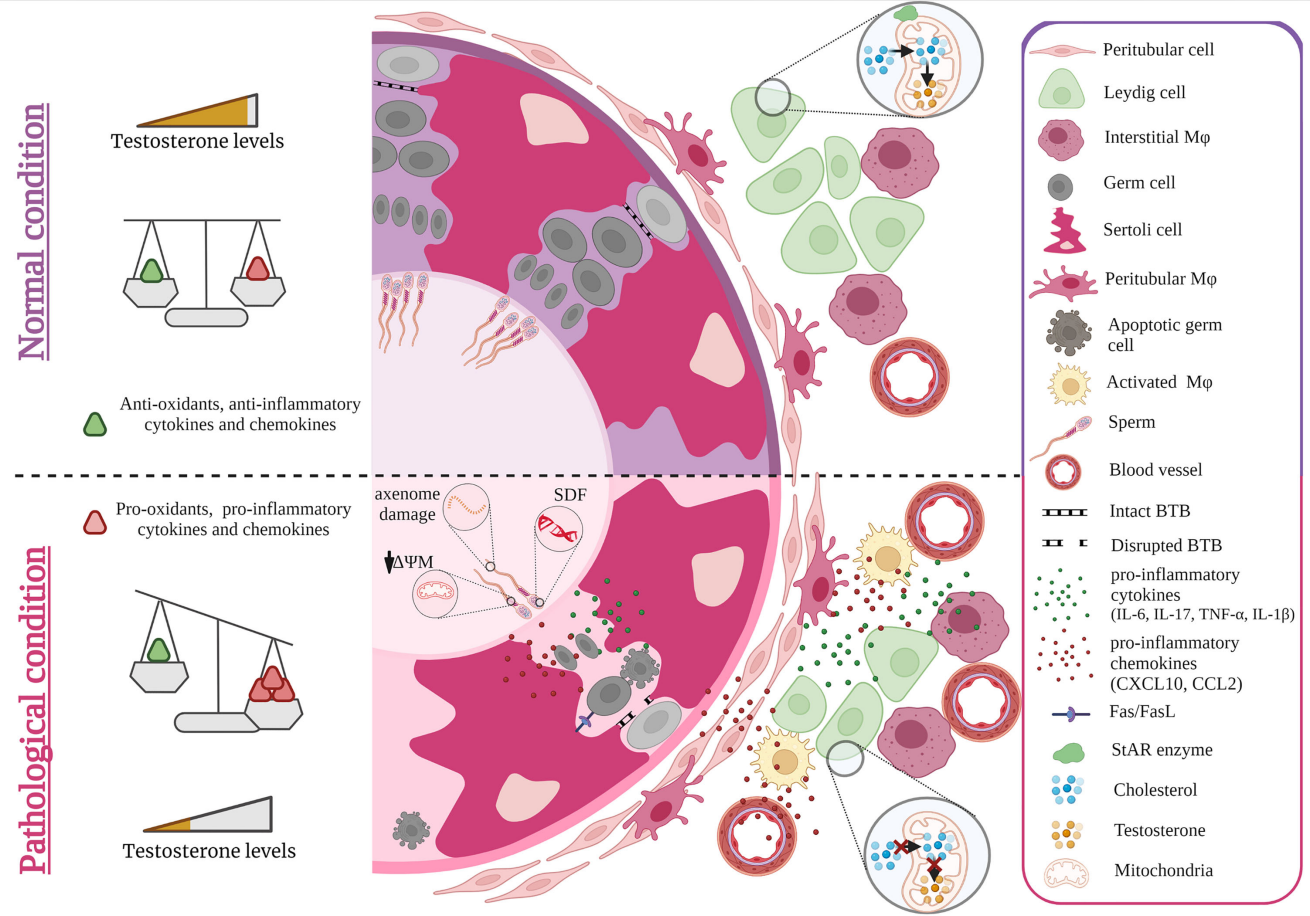
Effect of cytokines and oxidative stress on spermatogenesis and steroidogenesis. Under normal conditions, levels of anti- and pro-inflammatory cytokines, chemokines as well as anti-and pro-oxidants are balanced maintaining steroidogenesis and spermatogenesis. Sterile inflammation and microbial infection both cause an invasion of monocyte derived macrophages that together with increased production of pro-inflammatory cytokines such as IL-6, TNF-α, IL-17, IL-1β and chemokines such as CCL2, CXCL10 by resident testicular cells as well as recruited immune cells result in a shift towards higher levels of pro-inflammatory factors that negatively impact spermatogenesis and steroidogenesis. As a consequence, germ cell death and lower levels of androgens are observed e.g. by ROS diminishing the expression of StAR. ROS induced damage of spermatozoa also occurs during epididymal transit (axoneme damage, decreased mitochondrial potential = ΔΨM, sperm DNA fragmentation =SDF). Figure created with BioRender.com.

Reactive oxygen species (ROS) play an important role both in the maintenance of fertility in men, but also in pathological alterations of sperm parameters such as viability, motility, maturation, capacitation, hyperactivation and acrosome reaction (47). While ROS is required to combat pathogens and thus account for an effective anti-microbial immune response (48), supraphysiological levels of ROS, particularly for extended periods of time, can induce intense oxidative stress with toxic consequences for cells in general. In this regard, spermatozoa are particularly vulnerable due to their unique cytoarchitecture and biochemical characteristics (49–51). Spermatozoa possess a plasma membrane that is highly enriched in polyunsaturated fatty acids, particularly docosahexaenoic and arachidonic acids making them extremely susceptible to ROS-induced damage (52). Increased ROS production coupled with poor antioxidant capabilities in sperm can result in sperm DNA fragmentation (SDF) (**Figure 1**) (53). Elevated SDF alters the ultrastructure of sperm by leading to vacuolization in the nucleus along with other severe sperm morphological abnormalities that altogether can hinder fertilization by adversely affecting hyperactivation, capacitation and acrosome reaction (54). In this light, it is not surprising that SDF was reported in couples with unexplained recurrent pregnancy loss (55). Moreover, an initiation in the lipid peroxidation cascade can ultimately reduce sperm motility and viability owing to the fact that ROS-induced lipid peroxidation decreases mitochondrial membrane potential with concomitant structural damage in the adjacent axoneme (56, 57). The generation of lipid peroxidation products, particularly lipid aldehydes such as 4-hydroxynonenal (4-HNE), can negatively influence sperm motility as 4-HNE can bind to the dynein heavy chain in the sperm tail and to protein kinase anchoring protein 4 (AKAP4) in the sperm fibrous sheath (51) (**Figure 1**). In developing germ cells, oxidative stress can mediate cell death *via* several apoptotic pathways including activation of death receptors (Fas and TNFR1) and mitochondrial pathways (caspase 9) (58–60). The increased co-expression of Fas and FasL in germ cells implies that cell death *via* the Fas/FasL-mediated apoptotic signal transduction pathway could occur *via* autocrine and/or paracrine mechanisms (59). The susceptibility of germ cells to apoptosis *via* Fas/FasL could be regulated by Sertoli cells when the intracellular death domain of Fas reacts with FasL receptors on Sertoli cells (61, 62). Activated macrophages also play a role in the apoptosis of germ cells by releasing the stress response protein HMGB1 in response to inflammation-induced oxidative stress (**Figure 1**). In turn, HMGB1 causes germ cell death by inducing a decrease in anti-apoptotic Bcl-2 levels and a concomitant increase in pro-apoptotic Bax protein levels, cytochrome c and caspase 3 activity (63).

Alongside apoptosis, autophagy was reported as a pathway involved in disruption of spermatogenesis. In this context, increased expression of autophagy-related gene 7 (Atg7) was observed in spermatocytes after heat treatment of mice (64). The knockdown of Atg7, a factor required for formation of autophagosomes (65), *via* siRNA injected into the seminiferous tubules of these mice led to significant protection against heat-induced autophagy that was accompanied with decreased rates of germ cell apoptosis (64).

Intense oxidative stress can also affect Leydig cell steroidogenesis eventually leading to infertility. ROS can disturb Leydig cell mitochondria in diminishing the expression of steroidogenic acute regulatory protein (StAR) which in turn can decrease mitochondrial transport of cholesterol and consequently reduces synthesis of androgens (66, 67). This negative influence on steroidogenesis was reported to be a result of oxidative stress-induced activation of the p38 MAPK protein (68). C-Jun, a further stress responsive MAPK subfamily member, was also shown to be involved in suppressing the expression of steroidogenic enzymes as ROS mediated signaling upregulation of c-Jun inhibits Nur77 transactivation (69). Orphan nuclear receptors like Nur77 are known to be key transcriptional factors regulating the gene expression of steroidogenic enzymes (70, 71). Moreover, steroidogenesis can be downregulated in a paracrine fashion. This is elicited by TNF-α released by activated macrophages which addresses the TNF-α receptor TNFR1 expressed on neighboring Leydig cells. This leads to Leydig cell apoptosis and to activation of p38 MAPK signaling pathway resulting in decreased serum testosterone levels (72).

### Paracrine Influence of Cytokines, Chemokines and Growth Factors on Spermatogenesis and Steroidogenesis

Signaling molecules especially cytokines and growth factors and their receptors are widely produced by testicular cells. These signaling molecules play crucial roles in normal testis development and function when expressed at physiological levels, whereas increased levels can lead to disturbed organ function (73, 74). As an example, the activation of toll-like receptors (TLR) following binding of microbial pathogen-associated molecular patterns (PAMPs) and endogenous ligands such as alarmins (which are released during tissue damage) can initiate a cascade of signal transduction pathways which ultimately can culminate in the secretion of a range of signaling molecules including pro-inflammatory cytokines TNF-α, interleukin (IL)-1β and IL-6 in addition to chemokines (CXCL8 and CXCL10) (75) that all act in a paracrine fashion. Pathological consequences are indicated by neutralization of TNF-α in conditioned media of testicular macrophages, which results in decreased apoptosis of germ cells (74). Furthermore, murine *Tnf-α*^-/-^ Sertoli cells were protected from MuV-induced down-regulation of occludin and zonula occludin-1 thus safeguarding the integrity of the BTB. Inhibition of TNF-α production by the immunomodulatory drug pomalidomide in MuV infected Sertoli cells also prevented the disruption of the tight junction integrity of the BTB. Similar observations were made *in vivo* where TNF-α deficiency prevented the MuV induced disruption in the BTB and loss in spermatids (76).

TNF-α can also induce the production of CXCL10 in Sertoli cells in an autocrine manner, which can in turn induce apoptosis of germ cells *via* caspase-3 activation after binding to CXCR3 on these cells. As a control, the experimental deletion of the genes for CXCL10 or TNF-α in a co-culture of germ cells and Sertoli cells inhibits MuV-induced germ cell apoptosis (77). To add, CXCL10 and another chemokine ligand, CCL2, which is produced by Sertoli cells, Leydig cells and testicular macrophages in response to inflammation could recruit leukocytes resulting in a negative impact on spermatogenesis (**Figure 1**) (78). The role of a dysregulated CCL2/CCR2 axis on spermatogenesis was clearly shown in *Ccr2*^−/−^ mice that were protected from germ cell loss otherwise seen in acute bacterial epididymo-orchitis (21) TNF-α can also lead to elevated expression of activin A - a member of the transforming growth factor-β (TGFβ) family of cytokines - in Sertoli cells (**Figure 1**). Inhibiting activin A *in vivo* by elevating circulating levels of its antagonist follistatin reduced the overall severity of EAEO, associated germ cell loss and fibrotic damage (79). Further credence of a negative role of upregulated pro-inflammatory cytokines on spermatogenesis is derived from *in vivo* and *in vitro* experiments (74, 80–82). Testicular injection of IL-6 or IL-17A induced germ cell sloughing and disruption of the integrity of the BTB, a finding corroborated *in vitro* when murine Sertoli cells cultured with excess IL-6 or IL-17A exhibited a disrupted BTB integrity and permeability concomitant with a decrease in transepithelial electrical resistance that was associated with changes in the distribution of tight junction protein expression (occludin, claudin 11) (81, 83) (**Figure 1**). IL-6 can also directly induce apoptosis of germ cells *in vitro* (74, 84). Infection with Sars-Cov-2 was shown to increase the levels of pro-inflammatory cytokines mainly IL-6, TNF-α, IL-1β and this was accompanied with disruption in the expression of junctional proteins (occludin, claudin-11, connexin-43) along with decreased numbers of Sertoli cells and decreased sperm counts (85–87).

Increase in the aforementioned pro-inflammatory cytokines and chemokines can also negatively influence the ability of Leydig cells to synthesize testosterone mainly by acting as repressors of steroidogenic enzyme gene expression (88–90). TNF-α and TGF- β were found to be implicated in disrupting steroidogenesis directly *via* the competitive inhibitory action of NF-κB subunits on the transactivation of Nur77 and other orphan nuclear receptors (88, 91–93). Activated macrophages, which are physically interacting with Leydig cells, were shown to produce pro-inflammatory cytokines such as IL-1 and TNF-α that can inhibit Leydig cell steroidogenesis (66). In this co-culture setting of Leydig cells with activated testicular macrophages (via lipopolysaccharide stimulation), mRNA expression of steroidogenesis related genes (SF1, StAR and 3β-HSD) was inhibited (94). Moreover, IL-1β added to murine Leydig cells can induce the expression of CCL2, which in turn can decrease steroidogenic enzymes such as CYP17A1 and induce apoptosis as evidenced by cleaved caspase-3. This effect was also documented in human Leydig cells (95). Overexpression of another chemokine -CXCL10- in murine tumor Leydig cells also inhibit StAR expression and decrease cAMP-induced progesterone synthesis in a paracrine fashion (77).

## Significance and Conclusion

Cytokines and chemokines play an important role in the regulation of normal testicular function. They display direct paracrine effects on spermatogenic and Leydig cells that in the case of an upregulation during inflammatory episodes can impose harmful consequences. However, a degree of caution is necessary as a considerable amount of data relies on *in vitro* studies using isolated cells. Moreover, definitive functions of pro-inflammatory factors are difficult to determine as their action is context dependent and influenced by other mediators acting at the same target cell. Research harvesting breakthrough technologies like scRNA-seq and spatial transcriptomic is just about to unravel the overlap of the immune and testicular system and how they are linked in normal and pathological condition.

## Author Contributions

AM designed the outline of the manuscript. Writing and final editing was performed by all authors. Figure design was done by HH. All authors contributed to the article and approved the submitted version.

## Funding

This work was supported by a GRK 1871/2 International Research Training Group Giessen-Monash grant on ‘Molecular pathogenesis of male reproductive disorders’ (HH, SB, MF, AM) and a project grant BH 93/1-4 (awarded to SB) both from the Deutsche Forschungsgemeinschaft.

## Conflict of Interest

The authors declare that the research was conducted in the absence of any commercial or financial relationships that could be construed as a potential conflict of interest.

## Publisher’s Note

All claims expressed in this article are solely those of the authors and do not necessarily represent those of their affiliated organizations, or those of the publisher, the editors and the reviewers. Any product that may be evaluated in this article, or claim that may be made by its manufacturer, is not guaranteed or endorsed by the publisher.
